# Supplementary material for: Molecular Characterization of the First Alternavirus Identified in Fusarium oxysporum
Source: Viruses. 2021 Oct 8;13(10):2026. doi: 10.3390/v13102026 (PMC8538667; doi:10.3390/v13102026)
Supplement: Supplementary file 1 [file viruses-13-02026-s001.zip › Figure S1.pdf]

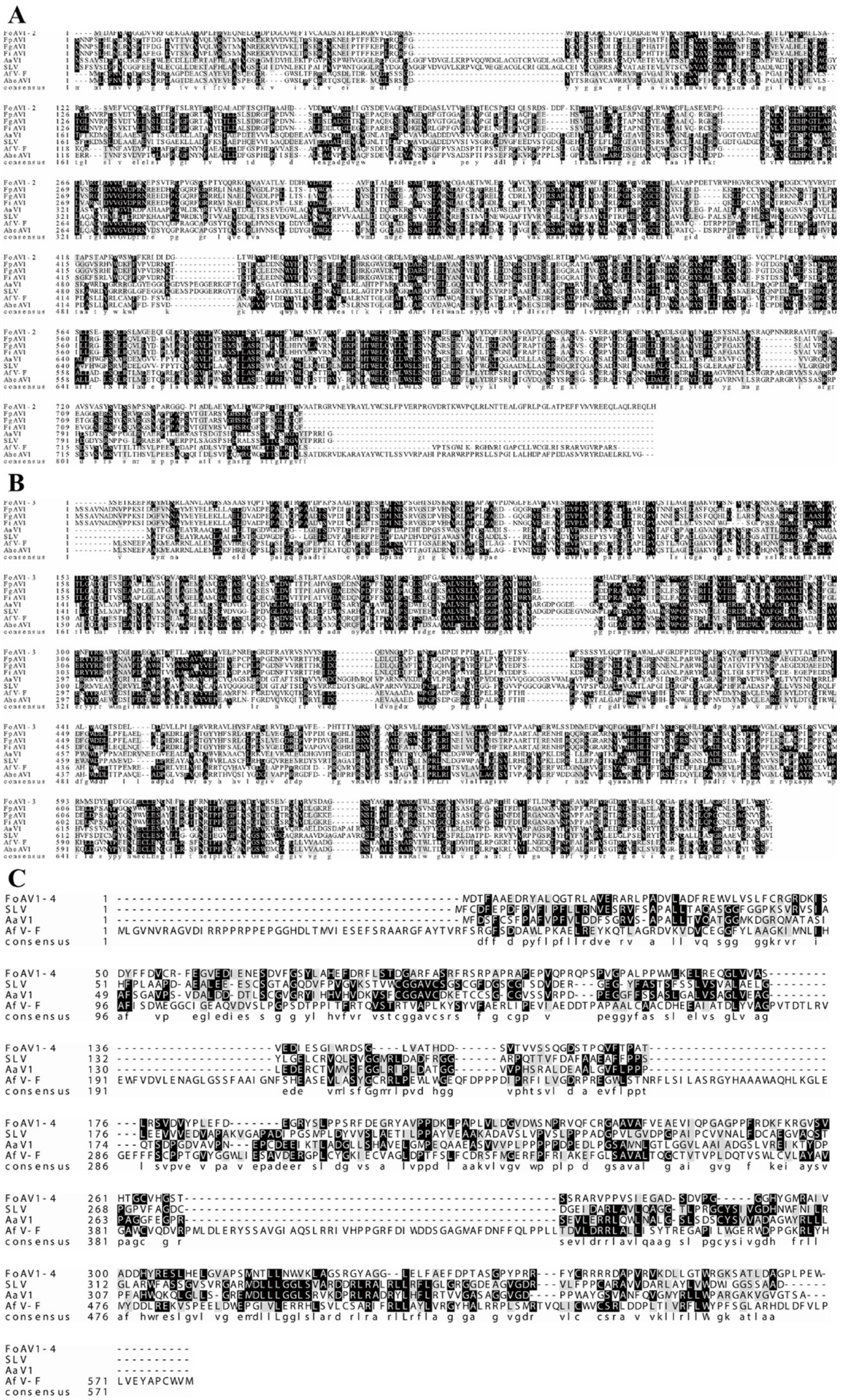

Figure S1. Multiple alignment of the ORF2(A), ORF3(B) and ORF4(C) amino acid motifs encoded by FoAV1 and other Alternaviridae family members, respectively.
