# Supplementary material for: Molecular Characterization of the First Alternavirus Identified in Fusarium oxysporum
Source: Viruses. 2021 Oct 8;13(10):2026. doi: 10.3390/v13102026 (PMC8538667; doi:10.3390/v13102026)
Supplement: Supplementary file 1 [file viruses-13-02026-s001.zip › Table S1.pdf]

Table S1. A list of primers used in this study.

Primer Name	Oligonucleotide sequence (5′- 3′)	Usage
RACE3RT	CGATCGATCATGATGCAATGCNNNNNN	For initial sequence cloning
RACE3	CGATCGATCATGATGCAATGC	
FoAV1-1F	ACTGCTAAGTGTCTCTCCCAATCCT	
FoAV1-1R	CAGGGAACAGACCAAACAAAGAAAG	
FoAV1-2F	TGACGGTTGTGGATGGATCTTC	
FoAV1-2R	ATACAGCAAACGCCTGGAAAGAGT	
FoAV1-3F	CGCCTTGTTTCTGTGCTTGCT	
FoAV1-3R	CACGCACAATACCCATCTCCG	
FoAV1-4F	TGGGGATGGATACTTTTGCTGCT	
FoAV1-4R	GACCGCCGCAACATATTCAAAA	
pC2	CGATCGATCATGATGCAATGC	For terminal sequence cloning
pC3-T7loop	p-GGATCCCGGGAATTCGGTAA TACGACTCACTATATTTTATAGT GAGTCGTATTA-OH	
FoAV1-1-A1	TGTGGCTCTTGGGTTGGT	
FoAV1-1-A2	ACAGAATCAACCACCTCACAAG	
FoAV1-1-S1	TGTCAACCTGGATGCGTAATG	
FoAV1-1-S2	CGTGGGTAAAGACATCAACTGG	
FoAV1-2-A1	CCTTTTCCCCGAACCTCACAA	
FoAV1-2-A2	ATCCATCCACAACCGTCAGG	
FoAV1-2-S1	CTTTATTGGTGTTCCCTTTCTCCG	
FoAV1-2-S2	GCCTACCTTTATTGGTGTTCCCTT	
FoAV1-3-A1	CTTCCGTGACAACCAAGAGCAT	
FoAV1-3-A2	TGGAACGGACCTGCCGACCCTGACA	
FoAV1-3-S1	AGACAGCGGCGAGGGTGAAAGC	
FoAV1-3-S2	ATAACAGACCTGATCCTTCGCAATA	
FoAV1-4-A1	ACGGCAGAACAGACTCACCAA	
FoAV1-4-S1	TTTGAATATGTTGCGGCGGT	
FoAV1-4-S2	CATGAACACTCTGCTTAACTGGGT	
BH19-1-YZ-F	GACAATGCTCAATATGCTGCTAAAG	Specific primers of the dsRNA1 segment of FoAV1
BH19-1-YZ-R	TCTTTCCACACCTTATGCGTTACT	
BH19-2-YZ-F	TGAGGTTCGGGGAAAAGGGT	Specific primers of the dsRNA2 segment of FoAV1
BH19-2-YZ-R	TGACAGTGTGAAGCGTAGTGCG	
BH19-3-YZ-F	GGAAGAGTTTCGTGCCTATATGGAC	Specific primers of the dsRNA3 segment of FoAV1
BH19-3-YZ-R	TTTCAGTTTATTGCCGCACGCT	
BH19-4-YZ-F	TTCAAATACCAGGTCCATCGCT	Specific primers of the dsRNA4 segment of FoAV1
BH19-4-YZ-R	GGTCATGCCACATCGCACAGT	
